# Sugarcane mosaic virus reduced bacterial diversity and network complexity in the maize root endosphere

**DOI:** 10.1128/msystems.00198-23

**Published:** 2023-06-29

**Authors:** Wenbo Liu, Xin Cui, Xinhai Wang, Cheng Shen, Lingfei Ji, Min Zhang, Ming Hung Wong, Jin Zhang, Shengdao Shan

**Affiliations:** 1 Key Laboratory of Recycling and Eco-treatment of Waste Biomass of Zhejiang Province, School of Environmental and Natural Resources, Zhejiang University of Science and Technology, Hangzhou, China; 2 State Key Laboratory of Agrobiotechnology and Key Laboratory of Pest Monitoring and Green Management-MOA, China Agricultural University, Beijing, China; 3 Department of Biology, University of York, Wentworth Way, York, United Kingdom; 4 Consortium on Health, Environment, Education and Research (CHEER), Department of Science and Environmental Studies, The Education University of Hong Kong, Tai Po, Hong Kong, China; Pacific Northwest National Laboratory, Richland, Washington, USA

**Keywords:** sugarcane mosaic virus, root endosphere, bacterial community assembly, plant-microbiota association

## Abstract

Sugarcane mosaic virus (SCMV) causes mosaic disease in crops such as maize and sugarcane by its vector—an aphid—and is transmitted top-down into the root system. However, understanding of the effects of the aphid-borne virus on root-associated microbes after plant invasion remains limited. The current project investigated maize root-associated (rhizosphere and endosphere) bacterial communities, potential interspecies interaction, and assembly processes in response to SCMV invasion based on 16S rRNA gene amplicon sequencing. SCMV was detected in the roots 9 days after inoculation, and leaf mosaic and chlorosis appeared. The SCMV invasion markedly reduced the α-diversity of endosphere bacteria compared with uninoculated controls (Mock). The connectivity and complexity of the bacterial co-occurrence network in the root endosphere decreased after SCMV invasion, implying that the plant virus may alter root endophyte-microbial interactions. Moreover, a signature that deviates more from stochastic processes was observed in virus-infected plants. Unexpectedly, the rhizosphere bacterial communities were rarely affected by the viral invasion. This study lays the foundation for elucidating the fate of the microbial component of the plant holobiont following aphid-borne virus exposure.

**IMPORTANCE:**

Biotic (e.g., soil-borne viruses) stress can alter root-associated bacterial communities, essential in maintaining host plant growth and health. However, the regulation of root-associated microorganisms by plant viruses from shoots is still largely unknown. Our results show that plant virus invasion leads to reduced and simpler inter-microbial communication in the maize endosphere. In addition, stochastic processes act on bacterial community assembly in both rhizosphere and endosphere, and bacterial communities in virus-invaded plant endosphere tend to shift toward deterministic processes. Our study highlights the negative effects of plant viruses on root endophytes from the microbial ecology perspective, which may be microbially mediated mechanisms of plant diseases.

## INTRODUCTION

Virus attacks are a common source of plant diseases leading to losses in crop production. Sugarcane mosaic virus (SCMV), a positive-sense single-stranded RNA (+ssRNA) virus (1), causes severe disease in monocotyledonous plant species such as maize and sugarcane (2). SCMV is vectored by aphids, which acquire and transmit SCMV by probing plant tissues. Along with the flow of nutrients, SCMV spreads quickly to the tissues of the whole plant resulting in chlorosis in emerging leaves (3) and stunted root growth (4). Despite our understanding of the adverse effects of SCMV on root growth, information on the influence of SCMV on root-associated microbiota remains scant.

The soil-root interface, the part of the soil around the living plant, boosts the transfer of nutrients, minerals, and energy between soil and roots (5). Inside both the rhizosphere and the endosphere, there are thousands of species of microbes. These microbes are part of the plant holobiont and play essential roles in nutrient uptake, abiotic stresses, and resistance to pathogens (6). When plants are stressed by pathogens, the root often initiates a “call for help” strategy to recruit beneficial microbes (7). For example, the endophytic microbiome mediates resistance to soil-borne viruses through the selective enrichment of microbiome species (8, 9). Further studies show that soil-borne viruses (e.g., wheat mosaic virus, *Furovirus* genus) alter the microbial community inside the endosphere/rhizosphere and enrich beneficial taxa such as *Streptomyces, Stenotrophomonas,* and *Bradyrhizobium* (10, 11). Soil-borne viruses usually infect plants directly from soil hosts (such as nematodes and fungi), which is a transfer route along the soil-rhizosphere-root system. Unlike the transmission of soil-borne viruses, the transfer of aphid-borne SCMV from shoots to roots occurs by transport through the vascular bundle (top-down process). Aphids as transmission agents make the SCMV spread on a broader range of time and distance than soil-borne viruses. In addition, differences in the transmission mechanisms of aphid-borne and soil-borne viruses may alter the responses of plants and associated microbiomes. However, whether or to what extent the transfer vector of SCMV will affect microbial community composition in the rhizosphere and endosphere is a question that remains to be answered.

In addition to potential beneficial groups of microorganisms, microbe-microbe (co-occurrence) interactions can also increase plant resistance to biotic stress (12). Microbial networks are powerful tools for analyzing microbial interactions. Healthy or strong plants usually steer a highly connected or complex microbial network (13 - 15). Although the microbial co-occurrence interactions are highly diverse, some types of interaction, such as secretion of antimicrobial compounds and competition for resources, may mitigate the growth of the virus or pathogen (12, 16, 17). For instance, changes in the microbial co-occurrence interactions in olive endosphere and rhizosphere induced susceptibility to the differential pathogen *Verticillium dahliae* (18). In turn, successful virus colonization in plants alters the co-occurrence network of microbes. Soil-borne virus (Chinese wheat yellow mosaic) invasion has recently been found to increase the complexity and connectivity of rhizosphere and root endophytic networks (10). However, the effects of the aphid-borne plant virus SCMV on microbial networks in the endosphere and rhizosphere remain poorly understood.

Understanding microbial community assembly processes from an ecological perspective can help to control or predict pathogen invasion (12, 19). Generally, the ecological processes of the microbial community assembly can be grouped into two classes, namely, deterministic and stochastic processes. These two processes simultaneously play essential roles in maintaining microbial community diversity and composition. Plant-associated microbial assembly is regulated not only by abiotic factors (e.g., climate and soil type) (20) but also by biotic stress (e.g., herbivorous insects and pathogen invasion) (21, 22). Viruses are biotic stress factors that have received little attention in regard to their impact on root-associated microbiome assembly. A recent study reveals that soil-borne wheat mosaic disease strengthens bacterial deterministic processes (23), but there are knowledge gaps in the understanding of the impact of aphid-borne viruses (e.g., SCMV) on the assembly processes of the microbial community, especially in the endosphere.

Given the importance of microbial community interactions and assembly processes, deciphering rhizosphere and endosphere communities following SCMV invasion may provide novel insights into the plant microbiome dysbiosis from the plant-virus-microbe perspective. Maize, a vital energy and food crop, was studied here because it is susceptible to SCMV virus damage. Hence, we artificially inoculated maize with the SCMV virus and determined the rhizosphere and endosphere bacterial community composition, interactions, and assembly processes. We hypothesized that (i) rhizosphere and endosphere bacterial communities are affected by virus invasion, (ii) inoculation with the virus reduces rhizosphere and endosphere bacterial network relationships and complexity due to bacterial dysbiosis, and (iii) bacterial assembly will tend to be more deterministic following virus infection because of biotic stress. This study aimed to fill the knowledge gap regarding the ecological processes of soil bacteria and to provide a foundation for controlling the invasion of SCMV in sustainable agriculture.

## RESULTS

### Plant characteristics after SCMV inoculation

**Fig 1:**
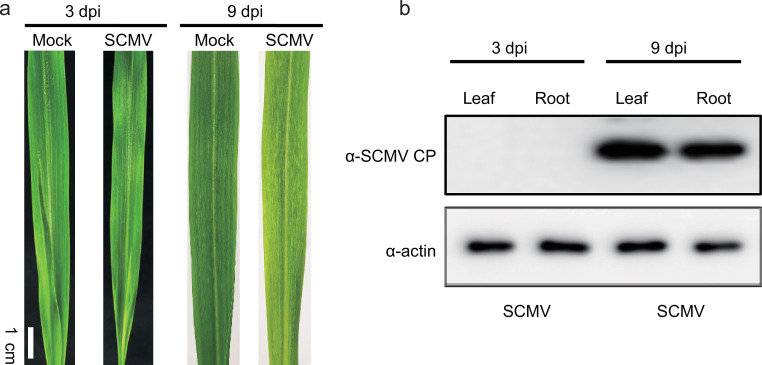
Symptoms of sugarcane mosaic virus (**a**) and western blotting of the leaf and root (**b**) after infection for 3 and 9 days. dpi, day post inoculation; SCMV CP, SCMV coat protein.

The color and characteristics of plant leaves changed significantly 3 days after inoculation (Fig. 1a). Still, the western blotting indicates no virus expression in leaves and roots at that time (Fig. 1b). The relative expression of SCMV in leaves appeared to increase but not significantly (Fig. S1a). Nine days after inoculation, the symptomatic leaves in the SCMV invasion treatment had many irregular yellow and green mosaics, stripes, or stripes alternating with parallel veins, which were more visible in sunlight. The relative abundance of the virus in the SCMV treatment in leaves was more than 300 times that in the control (Fig. S1a). In addition, the aboveground biomass did not change significantly with or without the virus (Fig. S1b). The protein of SCMV in the roots was observed after 9 days, indicating that the virus entered the roots from the aboveground parts after 9 days. The aim was to detect changes in bacterial communities in the rhizosphere and endosphere after virus invasion, therefore the following analysis focuses on their status 9 days after inoculation.

### Bacterial α- and β-diversity in the rhizosphere and endosphere

**Fig 2:**
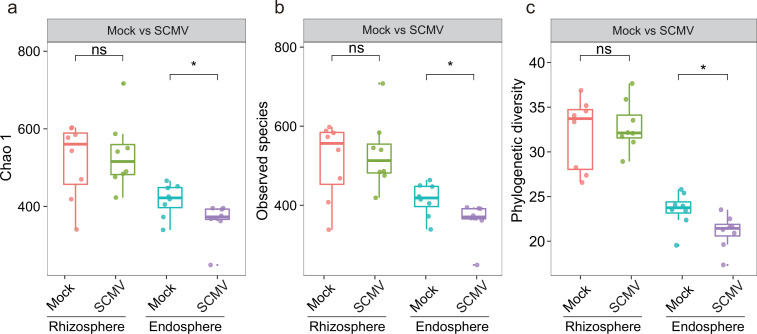
The bacterial α-diversity of the rhizosphere and endosphere in different treatments after infection for 9 days, comprising the Chao 1 (a), observed species (b), and phylogenetic diversity (c). Box and whisker plots represent the median and 25th–75th percentile (*n* = 8); ns, no significant difference; *: *P* < 0.05.

**Fig 3:**
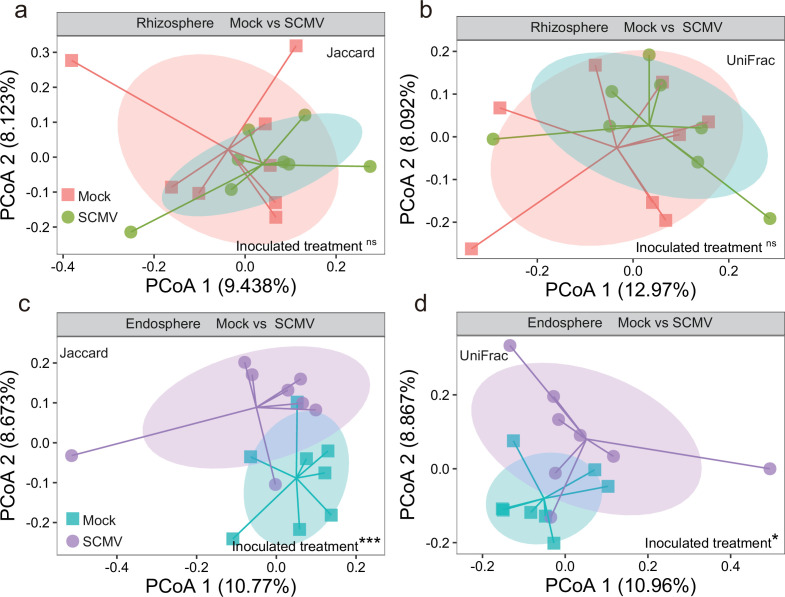
The bacterial β-diversity in the rhizosphere (**a and b**) and endosphere compartment (**c and d**) after infection for 9 days based on Jaccard distance (**a and c**) and unweighted UniFrac distance (**c and d**); ns, no significant difference; **: *P* < 0.01; and ***: *P* < 0.001.

The bacterial communities in the endosphere with SCMV displayed a lower diversity across all α*-*diversity metrics (Chao 1, observed species, and phylogenetic diversity) than those in the control, but no significant differences occurred in α-diversity indices between SCMV and Mock in the rhizosphere (Fig. 2). This indicates that virus invasion can reduce the bacterial richness in the endosphere and not the rhizosphere. Principal coordinate analysis (PCoA) based on Jaccard and unweighted UniFrac distances was conducted to determine the difference in bacterial communities between SCMV and Mock in endosphere and rhizosphere soil (Fig. 3). There was no significant change in the composition of the bacterial community in the rhizosphere with or without virus inoculation (Fig. 3a and b). Interestingly, the composition of the bacterial community changed substantially in the endosphere (Fig. 3c and d). Therefore, the bacterial richness [observed amplicon sequence variants (ASVs)] at different phylum levels was explored (Fig. S2). The entry of the virus into the roots did not affect the richness of the rhizosphere microorganisms at the phylum level. However, virus inoculation reduced the richness of Alphaproteobacteria, Betaproteobacteria, Actinobacteria, Bacteroidetes, and Acidobacteria in the endosphere. This indicates that some endosphere microorganisms were eliminated after inoculation.

### Shared and unique bacterial species between the rhizosphere and the endosphere

**Fig 4:**
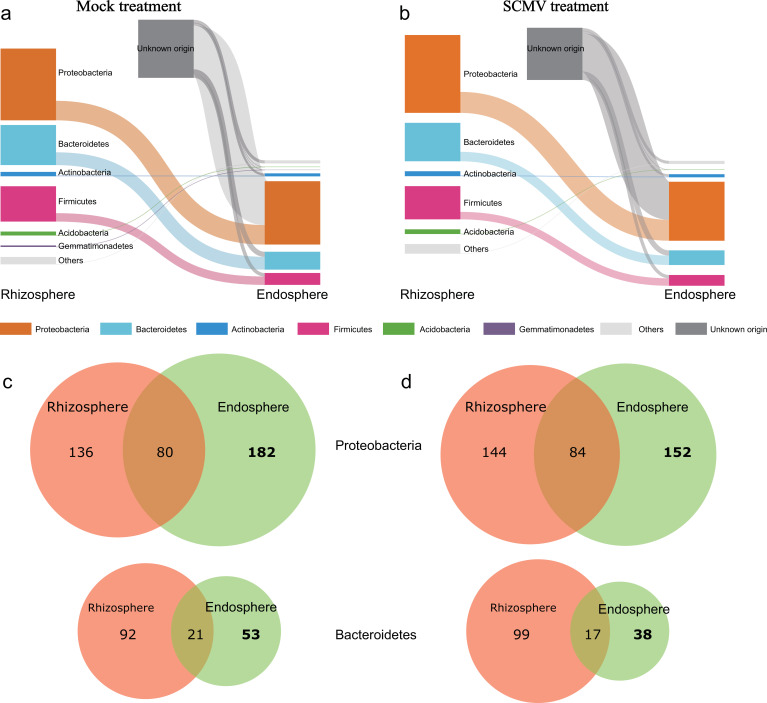
Sankey diagram showing changes in bacterial communities in the rhizosphere and endosphere in Mock control (**a**) and SCMV treatment (**b**). The bars on the left/right represent microorganisms present in the rhizosphere/endosphere, the flow in the middle represents the microorganisms shared by the rhizosphere and the endosphere, and the unknown represents the unique microorganisms in the endosphere. The Venn diagram shows that Proteobacteria and Bacteroidetes are shared at different compartments in Mock control (c) and SCMV treatment (d).

The shared microbial species between the rhizosphere and the endosphere was explored to understand the cause of the decrease in bacterial richness in endosphere after virus infection. However, Sankey plots found that the richness of common microorganisms in the rhizosphere and endosphere and the richness of unique bacteria in the rhizosphere did not change greatly (Fig. 4a and b). Except for the Proteobacteria and Bacteroidetes, the richness of ASV, which is unique to the microorganisms in the endosphere, did not change (Fig. 4c and d). The virus invasion reduced the number of ASVs belonging to Proteobacteria (from 182 to 152) and Bacteroidetes (from 53 to 38). This suggested that virus exposure might to some extent cause a decrease in the richness of specific root indigenous microbial taxa.

### Co-occurrence network of bacterial communities between control and SCMV treatment

**Fig 5:**
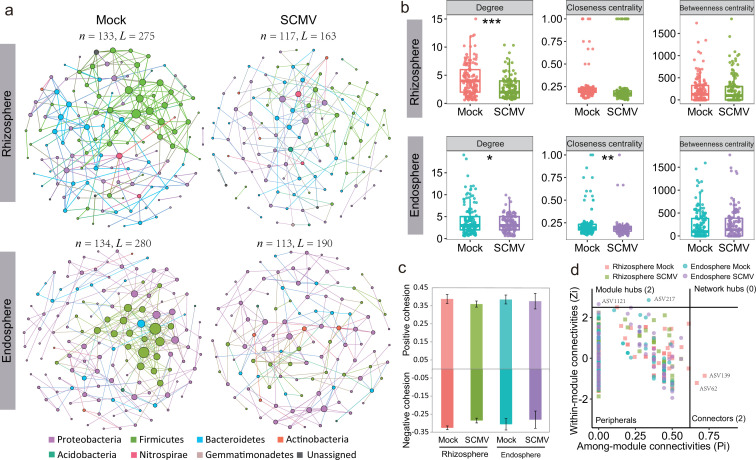
The bacterial correlation network in the rhizosphere and endosphere (**a**) after infection for 9 days. A link indicates a strong and significant correlation; *n*, node; *L*, link. The size of a node is proportional to the degree. The degree closeness centrality and betweenness centrality values of the nodes in the different networks (**b**); *, *P* < 0.05; **, *P* < 0.01; and ***, *P* < 0.001. The positive and negative cohesion of the bacterial correlation network (**c**). Classification of nodes to identify putative keystone species within the correlation network (**d**).

A co-occurrence network analysis identified potential bacterial interactions and niche sharing between the rhizosphere and the endosphere. Four bacterial co-occurrence networks were constructed among bacterial taxa at the ASV level. None of the four networks constructed were random networks (Table S1 to S4). In the rhizosphere or endosphere, the co-occurrence network map treated with Mock and SCMV differed to some extent (Fig. 5a). In terms of the rhizosphere, the degree of nodes in SCMV treatment was generally lower than that in Mock control (Fig. 5b). For root endophytes, the degree and closeness centrality (CC) values of nodes in SCMV treatment were significantly lower than those in Mock control. More and higher betweenness centrality (BC) values occurred in Mock control (Fig. 5b). Through the calculation of positive and negative cohesion it was found that compared with control, the cohesion decreased to varying degrees after SCMV treatment (Fig. 5c). Overall, virus inoculation reduced the complexity and cohesion of bacterial networks in endosphere.

The bacterial groups involved in the network interaction were mainly Proteobacteria, Firmicutes, and Bacteroidetes; the three groups in the rhizosphere network comprising about 32%, 30–38%, and 24%, respectively. In contrast, those in the endosphere network were about 66%, 19%, and 11%, respectively (Table S6). Most node degrees and BC values also decreased in the network inoculated with the virus, indicating that virus infection reduced the number of nodes in the center of the bridge and reduced the interaction. Zi-Pi plot results show that two connectors (ASV62 and ASV139) were found in the rhizosphere treated with Mock, while one module hub was detected in the rhizosphere treated with Mock and SCMV, and these were ASV217 and ASV1121, respectively (Fig. 5d).

### Assembly processes of bacterial communities between control and SCMV treatment

**Fig 6:**
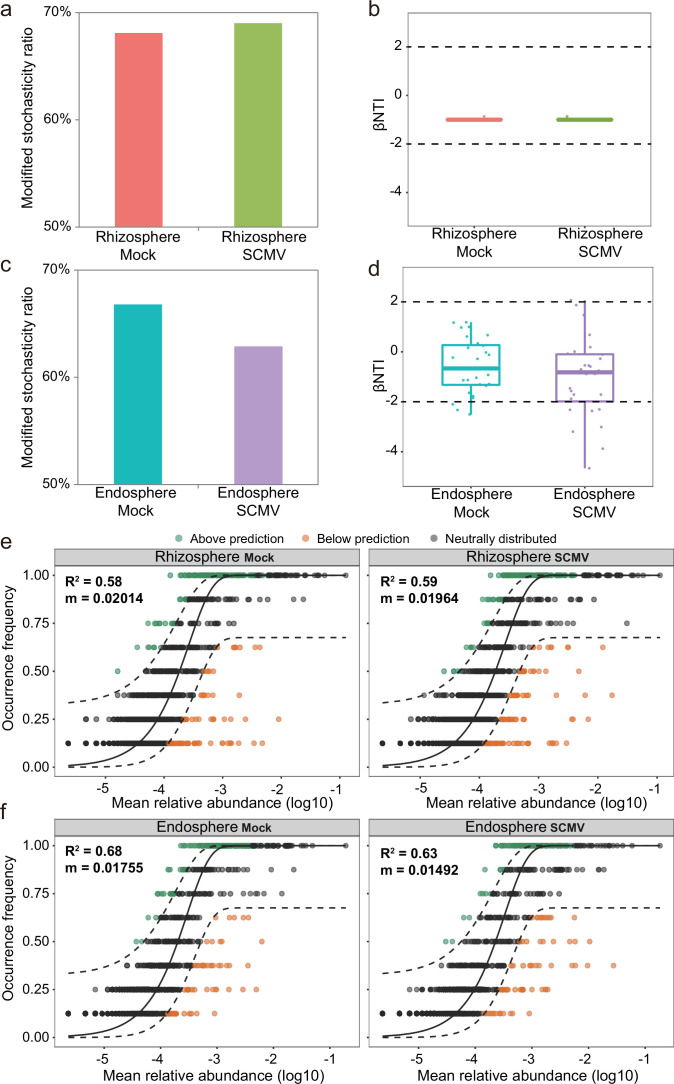
The assembly processes of the bacterial community in the rhizosphere (**a, b, e**) and endosphere (**c, d, f**) after virus infection. The modified stochasticity ratio of the bacterial community in the rhizosphere (**a**) and endosphere compartment (**c**) based on Jaccard distance. The β-NTI values considering presence/absence of ASV patterns (unweighted) in the rhizosphere (**b**) and endosphere compartment (**d**). Horizontal dashed black lines indicate upper and lower thresholds at β-NTI =  +2 and −2, respectively. Estimation of the neutral processes based on the Sloan neutral model community in the rhizosphere (**e**) and endosphere compartment (**f**). The parameters *R^2^* and *m* represent the goodness of fit and migration rate, respectively. The species that occur more and less frequently than predicted are shown in green and yellow, respectively. Dashed lines represent 95% confidence intervals, and the species falling within the confidence intervals are considered neutrally distributed.

The assembly processes of the bacterial community were assessed by three different models, relative importance of deterministic and stochastic processes [modified stochasticity ratio (MST) and β-nearest taxon index (β-NTI) value], and goodness of fit [Sloan neutral community model, (NCM)]. The MST values of the bacterial communities in the rhizosphere and endosphere were 68.9–69.9% and 64.2–67.7%, respectively (Fig. 6a and c). This suggests that stochasticity processes played a more critical role than deterministic ones in the bacterial community. There was no significant difference in MST between Mock and SCMV treatment in the rhizosphere bacterial community. However, in the endosphere, the MST of the bacterial community in SCMV treatment (64.2%) was lower than in the Mock control (67.7%). Based on the unweighted β-NTI value (only considering the presence/absence of ASV in the data set), the rhizosphere bacterial communities were dominated by stochastic processes (Fig. 6b). In contrast, the endosphere bacterial community in the SCMV treatment had more deterministic processes than that in the Mock control treatment (Fig. 6d). Also, the NCM of the bacterial communities was fitted in different treatments (Fig. 6e and f). Neutral processes also played an essential role in bacterial community assembly (*R^2^*, 0.58–0.68), as did the MST results. There was almost no change in the two treatments of *R^2^* in the rhizosphere, whereas the endosphere in SCMV had a smaller *R^2^* value (0.63) and estimated migration rates (*m*, 0.149) than in the control (*R^2^* = 0.68; *m* = 0.176). Similar trends of MST, β-NTI, and NCM were observed in endosphere, control, and SCMV treatment (Fig. 6). This indicates that bacterial taxa after virus inoculation had a higher tendency toward deterministic assembly processes and were more limited by dispersal. In short, plant virus invasion did not affect the assembly process of bacterial communities in the rhizosphere. However, inoculation with the plant virus in the endosphere biased bacterial communities more toward non-stochastic processes.

## DISCUSSION

SCMV is a positive-stranded RNA virus that can destroy the growth of crops (e.g., maize and sugarcane) within 12 days, potentially endangering global food and energy security. The +ssRNA virus that invades plants starts translation initially and then begins to be heavily transcribed at later stages (24). Therefore, the plant virus did not cause significant changes in maize characteristics or SCMV accumulation during the first 3 days of the invasion. Nine days after inoculation, SCMV-infected leaves showed overall yellowing with numerous yellow streaks, and the SCMV was greatly enriched in maize leaves and roots (Fig. 1; Fig. S1). These findings are consistent with a previous study (25). In addition to controlling plant virus invasion at the source, such as by molecular breeding, exploitation of resistant germplasm, application of virus-free plantlets, and agricultural strategies (1, 26), plant-associated microorganisms are also potentially important in resistance to pathogens or virus invasion. We have, therefore, focused on the severe period (9 days after inoculation) of maize disease to explore changes in the plant microbiome. This study has evaluated a little-noticed knowledge gap from a plant microbiome perspective: the impact of plant viral invasion on the composition, association, and assembly of the bacterial community in the root-associated (rhizosphere and endosphere) compartment.

### SCMV altered the richness and composition of bacterial communities in the endosphere

The rhizosphere is an important interface between soil and plant. It has a favorable environment (e.g., high in nutrients and energy) for microbial colonization (27), resulting in higher α-diversity than within the endosphere. The current study assesses how plant viruses affect the α-diversity and community composition of bacteria in the rhizosphere and endosphere. SCMV reduced bacterial richness in the maize endosphere but did not affect the rhizosphere (Fig. 2), and this partially supports our first hypothesis. Some studies have shown that soil-borne viruses or pathogens increase rhizosphere bacterial diversity (10, 28) and recruit beneficial microorganisms, thus initiating pathogen-resistant mechanisms. Varied responses to virus invasion in different compartments may be related to niche differences (29), plant growth conditions, and virus transport pathways. The unaffected bacterial diversity in the rhizosphere may be due to the absence of changes in plant biomass or rhizosphere metabolites (14, 30). We may speculate that severely wilted plants may result in changes in root exudates that affect rhizosphere bacterial diversity at later stages of virus infection.

Endosphere bacteria are affected by viral attacks before the rhizosphere because SCMV is transmitted to the root through the vascular bundles (top-down transport) (31). A decline in endophytic bacterial diversity was observed in diseased or pathogen-containing plants (32, 33). A previous report showed that microbial diversity may be affected by the synthesis of stress metabolites (including reactive oxygen species or phytoalexins), stress signals, and changes in bacterial habitats (34). Unfortunately, we did not measure the response of maize physiological metabolism to the SCMV (only chlorosis of leaves in the SCMV treatment), and the relationship between plant physiological metabolism and endosphere bacteria deserves attention in future research. In addition, the effects of plant viruses on bacterial α-diversity in the endosphere are distinct from the influences of bacterial pathogens. Bacterial pathogens affect endophytic diversity by directly or indirectly competing with endophytes for resources (nutrients and secondary metabolites) or available niches. Plant viruses indirectly affect the living environment of endosphere microorganisms by destroying host plant cells. For example, SCMV disturbs the structure and function of chloroplasts (35) and dramatically represses photosynthesis and metabolism at both transcriptional and translational levels (25).

The bacterial community composition of the endosphere was greatly influenced by SCMV invasion and, simultaneously, the rhizosphere was unaffected (Fig. 3). The maize rhizosphere and endosphere microbial communities consisted mainly of Proteobacteria, Bacteroidetes, Firmicutes, and Actinobacteria, in agreement with previous studies (36, 37). In the endosphere bacterial community, plant virus invasion reduced the richness of Alphaproteobacteria, Betaproteobacteria, Bacteroidetes, and Actinobacteria. These are copiotrophic microorganisms, a gathering of endophytes preferring a nutrient-rich environment. Alphaproteobacteria and Betaproteobacteria have often been defined as plant growth-promoting microorganisms, some of which have nitrogen fixation or symbiotic functions (e.g., Neorhizobium and Rhodobacter) (38). Some exceptions are Alphaproteobacteria/Betaproteobacteria, including *nod* genes, which are difficult to spread to symbionts (39). Decreased Bacteroidetes following viral invasion may reduce plant carbon metabolism as they may be degraders of complex biopolymers (40). Some species belonging to Proteobacteria and Actinobacteria, such as *Pseudomonas*, are affected by secondary metabolites such as benzoxazinoids released by maize roots (41). These findings suggest that copiotrophic microbes in the endosphere may shift to an oligotrophic environment with virus invasion, which could possibly result in the loss of plant-microbiome synergistic function. Specifically, metagenomes can provide a more detailed description of the metabolic potential of microbial communities.

Most of the root endophytic microorganisms originate from the soil, rhizosphere, phyllosphere, and seed (42). Sankey and Venn diagrams were used to analyze the sharing of bacteria in the endosphere and rhizosphere to understand whether plant viruses lead to changes in root endophytes (Fig. 4). The phylum-level bacterial communities within both the endosphere and the rhizosphere did not change markedly after the viral invasion, indicating to some extent that the plant virus does not alter the shared bacterial richness between rhizosphere and endosphere. Considering that the phylum level contains many bacteria with different metabolisms, we cannot deny that the shared sublevel microbial taxa (e.g., order level) did not change. Interestingly, the virus reduced the richness of bacteria unique to the endosphere (“unknown origin” in Fig. 4a and b), which was dominated by Proteobacteria and Bacteroidetes (Fig. 4c and d). The “unknown origin” percentage in the endosphere may be contributed by water or air as potential environmental sources (43). The possibility that these endosphere bacteria are derived from seeds through vertical transmission cannot be ruled out (44). Plant viruses can affect stomatal development, which further affects transport drivers and alters the horizontal transmission of nutrients, water, and microorganisms (45), resulting in an altered bacterial richness of the endosphere. In addition, decreased bacterial colonization may also be due to bacteria failing to adapt to the virus-infected endosphere environment or niche after horizontal transfer into the endosphere.

### Lower network stability and more deterministic assembly process following SCMV invasion

Microorganisms in the natural environment form complex association networks rather than thriving in isolation. Microbial network analysis allows one to decipher the interactions among microorganisms from complex microbial communities. The present results highlight how SCMV invasion impacted the network structure of rhizosphere and endosphere microbiota. They further indicate that SCMV-infected plants harbored a significantly lower complexity network with fewer nodes, links, average degree, and average clustering coefficient than the control plants in line with our second hypothesis. An opposite conclusion emerged in a recent study (10) showing that soil-borne viruses increased the nodes and edges of the rhizosphere network due to increased rhizosphere nutrient status and bacterial diversity following infection with yellow mosaic disease (contrary to our result). This may be due to differences in transmission paths, infection processes, and host plant responses caused by the two distinct viruses (soil-borne wheat mosaic virus vs aphid-transmitted SCMV). Nonetheless, our evidence is derived from the calculation of cohesion values showing that the SCMV infection caused a decrease in cohesion. These outcomes are concerning, as diseases of aboveground plant parts can disrupt the interaction of belowground biota, reducing host fitness. Some previous studies have also found that belowground root-associated microbial networks are a considerable mechanism by which plants can resist invasion by pathogenic bacteria (7, 18). Microbial network stability is positively correlated with network complexity (46), and simple networks are more vulnerable to environmental perturbations (or biotic stress) than complex networks (47) because different microbial taxa can complement each other. It is noteworthy that root-associated networks of SCMV-infected plants, especially the endosphere, have a higher proportion of negative correlations than healthy plants (Table S5). SCMV occupies plant cells and represses photosynthetic and metabolic processes (25). Hence, the response of the microbial network to virus or pathogen invasion must always be considered, and the use of synthetic microbial flora may provide a valuable means of closing the knowledge gap in root microbiota defense against virus-triggered adversity (48, 49).

The present results show that Proteobacteria (copiotrophic) dominated the endosphere network interactions compared to the rhizosphere. Furthermore, there were fewer species of taxa at the phylum level involved in endosphere networks than in the rhizosphere. In addition, healthy plants harbored more nodes with higher values of degree or CC than SCMV-infected plants (Fig. 5). The loss of network connections may alter microbial interactions within local niches within the root, and it is possible that other microbes may occupy this local niche in order to avoid alterations in the microbiome or plant function. However, potential keystone microbes have been extensively identified in many network-related studies to determine the role of microbes in ecology and evolutionary biology (50, 51). Here, two module hubs (in the endosphere of controls and SCMV-infected plants, respectively) and two connectors (in the rhizosphere of controls) were identified as keystone bacteria within the four co-occurrence networks (Table S7). The two potential keystone genera ASV217 and ASV1121 (module hub) belonging to the genera Sphingobium and Flavitalea were detected in the endosphere, which has been shown to function by promoting plant growth (52, 53). Future attempts should be made to increase the stability of microbial networks and their ability to resist biotic stress from the perspective of plant growth-promoting bacteria.

Understanding the underlying assembly process of bacterial communities is also necessary to unravel the sustainability of agroecological systems under viral invasion. Consistent with our third hypothesis, three complementary models (MST, β-NTI, and NCM) revealed that bacterial communities of endosphere in healthy plants, not rhizosphere bacterial communities, were more prone to random or neutral assembly processes compared with virus-inoculated plants (Fig. 6). These findings imply that although the assembly process of bacterial communities in the rhizosphere and endosphere is dominated by stochasticity (NCM >50%; *R* ^2^ = 0.58–0.68) (54), virus invasion reduces the proportion of stochastic processes, leading to a shift toward deterministic processes. A consistent result was found in a recent study of soil-borne viruses (23), which shows that deterministic processes were greatly enhanced with increasing infection levels. These changes can be attributed to the availability of resources (55) and niche limitation (56), which indicate strong habitat filtration in the SCMV-infected endosphere. Another possible explanation is a reduction in bacterial migration rate (Fig. 6f) and an increase in competition among microorganisms (Table S5), ultimately leading to the failure of colonization of microorganisms with low adaptive capacity. Unfortunately, our study did not find the selective recruitment of potential pathogen-suppressing bacteria that could secrete antibiotics or preempt ecological niches. These may be unique and difficult-to-control features of aboveground plant viruses.

### Conclusions

Inoculation with a plant aboveground virus (top-down transport) revealed changes in the bacterial community in the endosphere and the rhizosphere from the perspective of community composition, species interactions, and community assembly. Invasion of roots by SCMV reduced the bacterial α-diversity in the endosphere, mainly of Proteobacteria and Bacteroidetes. However, SCMV did not alter rhizosphere bacterial community diversity between the rhizosphere and the endosphere. Under unfavorable biotic stress caused by virus invasion, the interactions between microorganisms tend to be fragile and straightforward, and community assembly shifted from more stochastic to deterministic processes. Overall, the results shed light on the adverse effects of viral invasion from a microbial ecology perspective. The invasion of plant viruses from aboveground impairs plant physiological characteristics and harms the microbial component of the plant holobiont.

## MATERIALS AND METHODS

### Plant material, virus inoculation, and sample collection

Seeds of maize (*Zea mays* L.) inbred line B73 were used in pot experiments. The soil was collected from an experimental field at China Agricultural University (40°01′ N, 116°17′ E). The SCMV-BJ strain (sugarcane mosaic virus in this study) was propagated in maize plants, and crude extracts from infected leaf tissues were used for further inoculation.

Pot experiments were conducted in a greenhouse at China Agricultural University to examine and verify the effects of SCMV on the bacterial community in the rhizosphere and endosphere. Two treatments were established, SCMV inoculation (0.1 g/1 mL, 30 µL) and Mock (control). When the first true leaf of 9-day-old maize seedlings developed, crude extracts from the SCMV-infected leaves (SCMV treatment) were used to inoculate the maize seedlings in a way that mimics transmission by aphids, as previously described (57). Maize seedlings of the same age inoculated with a phosphate buffer (PBS) were used as Mock-inoculated (Mock control) plants. Maize leaves and roots were collected 3 and 9 days after inoculation (dpi) to monitor SCMV coat protein (CP) gene expression and translation. Nine days after inoculation, root and rhizosphere soil samples were collected for DNA extraction.

### Total RNA extraction and gene expression analysis

Total RNA was isolated from maize leaf tissues with TRIzol Reagent according to its manual using RNase-free DNase I (TaKaRa Bio, Tokyo, Japan) to remove genomic DNA. cDNA was synthesized using 2 µg total RNA, random primers, M-MLV reverse transcriptase, and recombinant ribonuclease inhibitor (Promega, Madison, WI, USA) per 25 µL reaction. A Fast-SYBR Reaction Kit (CWBIO, Beijing, China) was used to conduct the quantitative reverse transcriptase-polymerase chain reaction using gene-specific primers designed by National Center for Biotechnology Information (NCBI) Primer-BLAST (https://www.ncbi.nlm.nih.gov/tools/primer-blast/). The primer pairs of SCMV CP are SCMV-CP-F (AAA GCC GTA GTG AAG CCG AA) and SCMV-CP-R (CGG CTT TCT TAG CAA GTG GC). The expression level of ZmUbi (ubiquitin) was determined and used as the internal control (ZmUbi-F: GCC AAG GAA GAA GTC GGT GT and ZmUBi-R: GTA GCG GCC CTT CTT CAA GT). Relative expression levels of the target genes were calculated using the 2^−△△CT^ method (58). All experiments were replicated at least three times.

### Western blotting

Total protein was extracted from the first systemically infected leaf tissue using an extraction buffer consisting of 220 mM Tris-HCl, 250 mM sucrose, 1 mM MgCl_2_, 50 mM KCl, and 10 mM β-mercaptoethanol. After incubation for 10 min, the extract was centrifuged twice for 10 min at 10,000 × *g* at 4℃ to pellet the protein. The proteins detected were visualized using the ECL Western Blot Detection System (Bio-Rad, Shanghai, China).

### Rhizosphere and endosphere DNA extraction, and sequencing of the 16S rRNA gene

Roots were shaken vigorously to remove the adhering soil to collect the endosphere sample. The roots were then washed with PBS twice (50 mL sterile flasks with 20 mL PBS for 20 min) to separate roots and rhizosphere soil (the residual soil was washed with the PBS solution). The washed roots were then sonicated for 10 min of 30 s cycles at 4,000 Hz to remove the remaining microorganisms on the root surface (59). The clean roots were grounded in liquid nitrogen and stored at −80°C for further treatment. The rhizosphere soil with the PBS solution was centrifuged at 11,000 × *g* for 5 min, and the supernatant was discarded. The rhizosphere soil fraction remaining was stored at −20°C for further treatment.

According to the manual, root and rhizosphere soil DNA was extracted using the FastDNA Spin Kit (MP Biomedicals, Irvine, CA USA). The quality of the DNA extracts was evaluated with a NanoDrop Spectrophotometer (NanoDrop Technologies, Wilmington, DE, USA). Bacterial 16S rRNA gene fragments (V5–V7) were amplified from the extracted DNA using primer sets 799F (5′-AAC MGG ATT AGA TAC CCKG-3′) and 1223R (5′-CCA TTG TAG TAC GTG TGTA-3′), as the primers 799F-1223R would amplify less plant host sequence than the primers 799F-1193R (data not shown). The DNA amplification and sequencing were done using an Illumina NovaSeq 6000 by Genesky Biotechnologies Inc. (Shanghai, China).

### Sequence processing of 16S rRNA gene

The QIIME2 analysis platform was used to process raw reads (https://qiime2.org). The first step was trimming the adaptor and primer sequences with the cutadapt plugin. The quality control and the ASVs identification (p-max-ee = 2.0, p-trunc-q = 2, and p-chimera-method = consensus) were then implemented using the DADA2 plugin (60). A Naive Bayes Classifier (Ribosomal Database Project, RDP version 11.5) was used to classify ASV representative sequences into taxonomic assignments. Each sample was then unified to 53,879 sequences depth for downstream analysis to eliminate the sequencing depth variation of different samples. The raw data were deposited in the SRA database of NCBI, and the biosample IDs are SAMN23169814—23169829 and SAMN23169838—23169853.

### Co-occurrence network construction

Bacterial co-occurrence networks were constructed using the Molecular Ecological Network Analyses Pipeline (50). Briefly, ASVs with an average relative abundance >0.05% and occurring in at least seven replicates were retained for network inference. The network construction was based on recommended parameters in data preparation and random matrix theory default settings. Bacterial co-occurrence networks were visualized with Gephi (v0.9.5). The network modules were characterized by greedy modularity optimization. The nodes inside modules were little associated with nodes outside. The topological properties of the nodes were calculated to assess the roles of ASVs in the networks by degree, CC, and BC. Furthermore, the nodes were characterized into four classes in light of the within-module connectivity (Zi) and among-module connectivity (Pi), including network hubs (Zi >2.5 and Pi >0.62), module hubs (nodes highly associated inside a module, Pi >0.62), connectors (i.e., nodes connecting various modules, Pi >0.62), and peripherals (Zi <2.5 and Pi <0.62) (50, 51).

By portraying positive or negative co-occurrences relationship independently, cohesion gives bits of knowledge into relationships among taxa brought about by both positive and negative species associations or additionally by both closeness and contrasts in the specialties of microbial taxa (61). Positive (range value from 0 to 1) and negative (from −1 to 0) cohesion with higher absolute values collectively represent more or stronger correlations. The larger negative cohesion values usually indicate more stable communities.

### The stochastic ratio and neutral model in community assembly

The MST, Sloan NCM, and β-NTI were used to evaluate the bacterial community assembly process. First, the MST, modified method as previously described (62), estimates that deterministic processes driving the community are more similar or dissimilar than the null expectation based on Jaccard distance using the “NST” package. According to the model, the microbial community assembly was more deterministic when the MST index was <0.5. In contrast, an >0.5 value of the MST index indicates that the community assembly is more stochastic. Second, the NCM, a neutral-theory-based approach (63), assumes that species are ecologically functionally equivalent, indicating that species may be randomly lost and then supplemented from the metacommunity via dispersal or replaced by other members of the local community (64). To explore the potential contribution of neutral processes, the relationship between the occurrence frequency of species and their relative abundance in the metacommunity was fitted using NLS model in R software (63). *R^2^* and *m* values represent the goodness of fit and bacterial migration rate. Third, β-NTI value was computed by null-model expectations (considering phylogenetic distance) (65, 66). β-NTI values (unweighted) were quantified without accounting for taxa relative abundances. Values of β-NTI >+2 or <−2 represent community turnover governed by deterministic process, while β-NTI values >+2 or <−2 were used as estimates of the stochastic process.

### Statistical analysis

All statistical analysis was conducted using the R software (http://www.r-project.org). t-Tests were employed using the function “t.test” to assess the difference in the α-diversity of the bacterial community, aboveground biomass, relative expression of SCMV CP, and characteristics of network nodes between Mock and SCMV treatment. The α-diversity of bacterial communities was determined by the Chao 1, and observed species and phylogenetic diversity were detected using the “vegan” and “picante” packages. PCoA was used to display the variation in bacterial communities based on Jaccard dissimilarity (vegan package) and unweighted UniFrac distance (GUniFrac package). Permutational analysis of variance was conducted to analyze the impact of virus inoculation on the bacterial community using the “adonis” function of the “vegan” package. Sankey graphs were plotted on an online website (https://sankeymatic.com). Other diagrams were created using the “ggplot2” package in the R platform. Raw data about plants are stored in the FigShare platform (https://doi.org/10.6084/m9.figshare.22698187.v1).

## SUPPLEMENTAL MATERIAL

Fig. S1 msystems.00198-23-s0001.tif Relative expression of SCMV CP in leaves (a) and aboveground biomass (b) after infection for three and nine days. Mock, uninoculated control; SCMV, sugarcane mosaic virus-inoculated treatment; ns, no significant difference; ***, p < 0.001.

Fig. S2 msystems.00198-23-s0002.tif The bacterial richness at phylum level in the rhizosphere (a, b) and endosphere (c, d) after infection for nine days; *, p < 0.05; **, p < 0.01; ***, p < 0.001.

Table S1 msystems.00198-23-s0003.docx Topological properties of the empirical network of the rhizosphere (control) and the associated random networks.

Table S2 msystems.00198-23-s0004.docx Topological properties of the empirical network of the rhizosphere (SCMV-inoculated) and the associated random networks.

Table S3 msystems.00198-23-s0005.docx Topological properties of the empirical network of the roots (control) and the associated random networks.

Table S4 msystems.00198-23-s0006.docx Topological properties of the empirical network of the roots (SCMV-inoculated) and the associated random networks.

Table S5 msystems.00198-23-s0007.docx The properties of rhizosphere and root bacterial networks in different treatments.

Table S6 msystems.00198-23-s0008.docx Percentages of microbial taxa participating in different networks.

Table S7 msystems.00198-23-s0009.docx Taxonomic information on keystone taxa in each network.

Supplemental legends msystems.00198-23-s0010.txt Legends to the supplemental figures and tables.

ASM does not own the copyrights to Supplemental Material that may be linked to, or accessed through, an article. The authors have granted ASM a non-exclusive, world-wide license to publish the Supplemental Material files. Please contact the corresponding author directly for reuse.
