# Supplementary figures and images for: Sugarcane mosaic virus reduced bacterial diversity and network complexity in the maize root endosphere
Source: mSystems. 2023 Jun 29;8(4):e00198-23. doi: 10.1128/msystems.00198-23 (PMC10469604; doi:10.1128/msystems.00198-23)

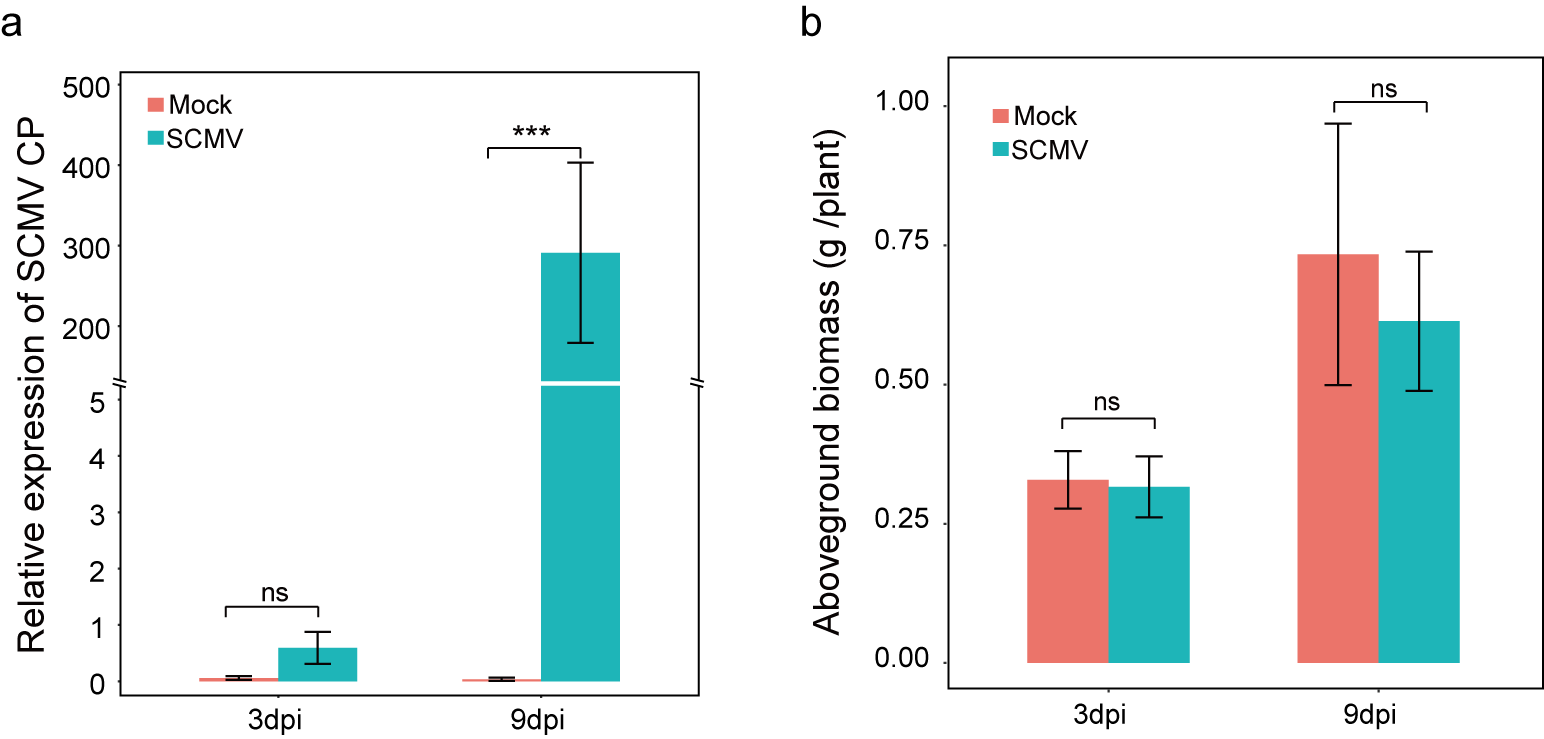

Supplement: Fig. S1 — Relative expression of SCMV CP in leaves (a) and aboveground biomass (b) after infection for three and nine days. Mock, uninoculated control; SCMV, sugarcane mosaic virus-inoculated treatment; ns, no significant difference; ***, p < 0.001. [file msystems.00198-23-s0001.tif]

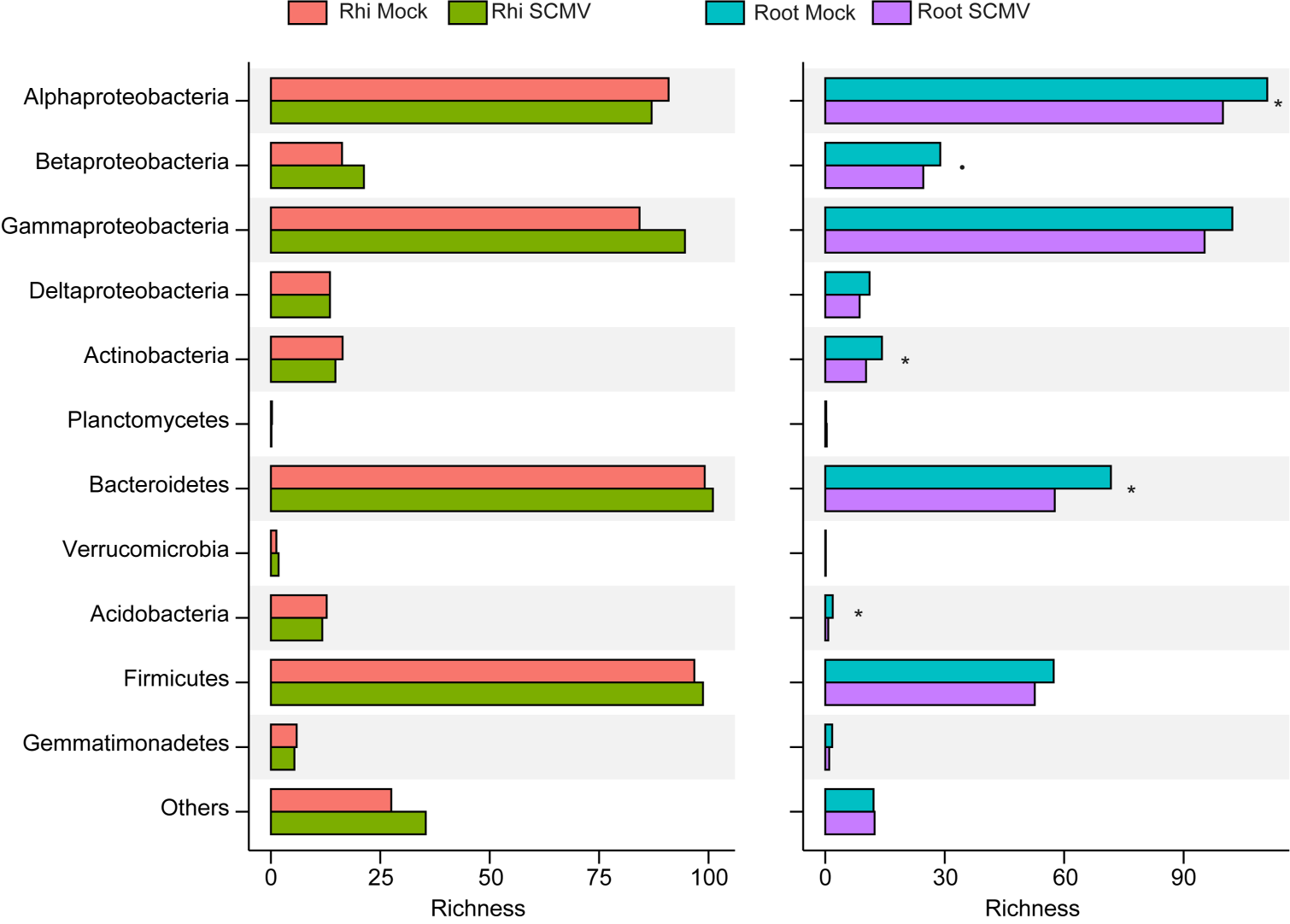

Supplement: Fig. S2 — The bacterial richness at phylum level in the rhizosphere (a, b) and endosphere (c, d) after infection for nine days; *, p < 0.05; **, p < 0.01; ***, p < 0.001. [file msystems.00198-23-s0002.tif]
