# Supplementary material for: Sugarcane mosaic virus reduced bacterial diversity and network complexity in the maize root endosphere
Source: mSystems. 2023 Jun 29;8(4):e00198-23. doi: 10.1128/msystems.00198-23 (PMC10469604; doi:10.1128/msystems.00198-23)
Supplement: Table S1 — Topological properties of the empirical network of the rhizosphere (control) and the associated random networks. [file msystems.00198-23-s0003.docx]

Table S1. Topological properties of the empirical network of the rhizosphere (control) and the associated random networks.

| Network Index | Empirical Network Index | 100 Random Networks Index |
| --- | --- | --- |
| Average clustering coefficient (avgCC) | 0.300 | 0.040 +/- 0.012 |
| Average path distance (GD) | 5.039 | 3.514 +/- 0.064 |
| Geodesic efficiency (E) | 0.257 | 0.327 +/- 0.004 |
| Harmonic geodesic distance (HD) | 3.885 | 3.063 +/- 0.038 |
| Centralization of degree (CD) | 0.084 | 0.084 +/- 0.000 |
| Centralization of betweenness (CB) | 0.176 | 0.114 +/- 0.018 |
| Centralization of stress centrality (CS) | 0.718 | 0.333 +/- 0.044 |
| Centralization of eigenvector centrality (CE) | 0.332 | 0.246 +/- 0.029 |
| Density (D) | 0.031 | 0.031 +/- 0.000 |
| Reciprocity | 1 | 1.000 +/- 0.000 |
| Transitivity (Trans) | 0.378 | 0.053 +/- 0.011 |
| Connectedness (Con) | 0.842 | 0.970 +/- 0.029 |
| Efficiency | 0.971 | 0.975 +/- 0.001 |
| Hierarchy | 0 | 0.000 +/- 0.000 |
| Lubness | 1 | 1.000 +/- 0.000 |
| Modularity(fast_greedy) | 0.653 | 0.457 +/- 0.010 |
