# Supplementary material for: Sugarcane mosaic virus reduced bacterial diversity and network complexity in the maize root endosphere
Source: mSystems. 2023 Jun 29;8(4):e00198-23. doi: 10.1128/msystems.00198-23 (PMC10469604; doi:10.1128/msystems.00198-23)
Supplement: Table S2 — Topological properties of the empirical network of the rhizosphere (SCMV-inoculated) and the associated random networks. [file msystems.00198-23-s0004.docx]

Table S2. Topological properties of the empirical network of the rhizosphere (SCMV-inoculated) and the associated random networks.

| Network Index | Empirical Network Index | 100 Random Networks Index |
| --- | --- | --- |
| Average clustering coefficient (avgCC) | 0.261 | 0.022 +/- 0.012 |
| Average path distance (GD) | 6.202 | 4.468 +/- 0.169 |
| Geodesic efficiency (E) | 0.217 | 0.268 +/- 0.007 |
| Harmonic geodesic distance (HD) | 4.611 | 3.734 +/- 0.096 |
| Centralization of degree (CD) | 0.063 | 0.063 +/- 0.000 |
| Centralization of betweenness (CB) | 0.241 | 0.192 +/- 0.041 |
| Centralization of stress centrality (CS) | 0.601 | 0.390 +/- 0.084 |
| Centralization of eigenvector centrality (CE) | 0.373 | 0.307 +/- 0.029 |
| Density (D) | 0.024 | 0.024 +/- 0.000 |
| Reciprocity | 1 | 1.000 +/- 0.000 |
| Transitivity (Trans) | 0.331 | 0.030 +/- 0.014 |
| Connectedness (Con) | 0.790 | 0.919 +/- 0.050 |
| Efficiency | 0.980 | 0.983 +/- 0.001 |
| Hierarchy | 0 | 0.000 +/- 0.000 |
| Lubness | 1 | 1.000 +/- 0.000 |
| Modularity(fast_greedy) | 0.757 | 0.604 +/- 0.013 |
