# Supplementary material for: Sugarcane mosaic virus reduced bacterial diversity and network complexity in the maize root endosphere
Source: mSystems. 2023 Jun 29;8(4):e00198-23. doi: 10.1128/msystems.00198-23 (PMC10469604; doi:10.1128/msystems.00198-23)
Supplement: Table S3 — Topological properties of the empirical network of the roots (control) and the associated random networks. [file msystems.00198-23-s0005.docx]

Table S3. Topological properties of the empirical network of the endosphere (control) and the associated random networks.

| Network Index | Empirical Network Index | 100 Random Networks Index |
| --- | --- | --- |
| Average clustering coefficient (avgCC) | 0.351 | 0.056 +/- 0.013 |
| Average path distance (GD) | 5.345 | 3.453 +/- 0.072 |
| Geodesic efficiency (E) | 0.248 | 0.332 +/- 0.004 |
| Harmonic geodesic distance (HD) | 4.028 | 3.014 +/- 0.040 |
| Centralization of degree (CD) | 0.113 | 0.113 +/- 0.000 |
| Centralization of betweenness (CB) | 0.156 | 0.152 +/- 0.019 |
| Centralization of stress centrality (CS) | 0.472 | 0.461 +/- 0.056 |
| Centralization of eigenvector centrality (CE) | 0.308 | 0.278 +/- 0.023 |
| Density (D) | 0.031 | 0.031 +/- 0.000 |
| Reciprocity | 1 | 1.000 +/- 0.000 |
| Transitivity (Trans) | 0.464 | 0.078 +/- 0.011 |
| Connectedness (Con) | 0.818 | 0.980 +/- 0.025 |
| Efficiency | 0.970 | 0.975 +/- 0.001 |
| Hierarchy | 0 | 0.000 +/- 0.000 |
| Lubness | 1 | 1.000 +/- 0.000 |
| Modularity(fast_greedy) | 0.595 | 0.447 +/- 0.011 |
