# Supplementary material for: Sugarcane mosaic virus reduced bacterial diversity and network complexity in the maize root endosphere
Source: mSystems. 2023 Jun 29;8(4):e00198-23. doi: 10.1128/msystems.00198-23 (PMC10469604; doi:10.1128/msystems.00198-23)
Supplement: Table S4 — Topological properties of the empirical network of the roots (SCMV-inoculated) and the associated random networks. [file msystems.00198-23-s0006.docx]

Table S4. Topological properties of the empirical network of the endosphere (SCMV-inoculated) and the associated random networks.

| Network Index | Empirical Network Index | 100 Random Networks Index |
| --- | --- | --- |
| Average clustering coefficient (avgCC) | 0.336 | 0.027 +/- 0.013 |
| Average path distance (GD) | 5.790 | 3.981 +/- 0.083 |
| Geodesic efficiency (E) | 0.231 | 0.294 +/- 0.004 |
| Harmonic geodesic distance (HD) | 4.332 | 3.399 +/- 0.046 |
| Centralization of degree (CD) | 0.060 | 0.060 +/- 0.000 |
| Centralization of betweenness (CB) | 0.246 | 0.134 +/- 0.024 |
| Centralization of stress centrality (CS) | 0.453 | 0.294 +/- 0.057 |
| Centralization of eigenvector centrality (CE) | 0.332 | 0.247 +/- 0.038 |
| Density (D) | 0.030 | 0.030 +/- 0.000 |
| Reciprocity | 1 | 1.000 +/- 0.000 |
| Transitivity (Trans) | 0.342 | 0.032 +/- 0.014 |
| Connectedness (Con) | 0.948 | 0.985 +/- 0.023 |
| Efficiency | 0.977 | 0.978 +/- 0.001 |
| Hierarchy | 0 | 0.000 +/- 0.000 |
| Lubness | 1 | 1.000 +/- 0.000 |
| Modularity(fast_greedy) | 0.745 | 0.530 +/- 0.014 |
