# Supplementary material for: Sugarcane mosaic virus reduced bacterial diversity and network complexity in the maize root endosphere
Source: mSystems. 2023 Jun 29;8(4):e00198-23. doi: 10.1128/msystems.00198-23 (PMC10469604; doi:10.1128/msystems.00198-23)
Supplement: Table S5 — The properties of rhizosphere and root bacterial networks in different treatments. [file msystems.00198-23-s0007.docx]

Table S5. The properties of rhizosphere and endosphere bacterial networks in different treatments.

| **Network property** | Rhizosphere (control) | Rhizosphere (SCMV) | Endosphere(control) | Endosphere (SCMV) |
| --- | --- | --- | --- | --- |
| **Total nodes** | 133 | 117 | 134 | 113 |
| **Total links** | 275 | 163 | 280 | 191 |
| **Positive link/total link** | **79.6%** | **71.2%** | **83.9%** | **60.2%** |
| **Negative link/total link** | **20.4%** | **28.8%** | **16.1%** | **39.8%** |
| **Average degree (avgK)** | 4.135 | 2.786 | 4.179 | 3.381 |
| **Average clustering coefficient (avgCC)** | 0.3 | 0.261 | 0.351 | 0.336 |
| **Average path distance (GD)** | 5.039 | 6.202 | 5.345 | 5.79 |
| **Centralization of degree (CD)** | 0.084 | 0.063 | 0.113 | 0.06 |
| **Centralization of betweenness (CB)** | 0.176 | 0.241 | 0.156 | 0.246 |
