# Supplementary material for: Sugarcane mosaic virus reduced bacterial diversity and network complexity in the maize root endosphere
Source: mSystems. 2023 Jun 29;8(4):e00198-23. doi: 10.1128/msystems.00198-23 (PMC10469604; doi:10.1128/msystems.00198-23)
Supplement: Table S6 — Percentages of microbial taxa participating in different networks. [file msystems.00198-23-s0008.docx]

Table S6. Percentages of microbial taxa participating in different networks.

| **Phylum** | **Rhizosphere (control)** | **Rhizosphere (SCMV)** | **Endosphere (control)** | **Endosphere (SCMV)** |
| --- | --- | --- | --- | --- |
| Proteobacteria | 31.58% | 33.33% | **65.67%** | **63.72%** |
| Firmicutes | 38.35% | 30.77% | 18.66% | 19.47% |
| Bacteroidetes | 24.06% | 24.79% | 11.94% | 11.50% |
| Actinobacteria | 1.50% | 3.42% | 2.24% | 3.54% |
| Acidobacteria | 0 | 2.56% | 0 | 0 |
| Nitrospirae | 1.50% | 1.71% | 0 | 0 |
| Gemmatimonadetes | 0 | 0.85% | 0 | 0 |
| Unassigned | 2.26% | 2.56% | 1.49% | 1.77% |
