# Supplementary material for: Sugarcane mosaic virus reduced bacterial diversity and network complexity in the maize root endosphere
Source: mSystems. 2023 Jun 29;8(4):e00198-23. doi: 10.1128/msystems.00198-23 (PMC10469604; doi:10.1128/msystems.00198-23)
Supplement: Table S7 — Taxonomic information on keystone taxa in each network. [file msystems.00198-23-s0009.docx]

Table S7. Taxonomic information on keystone taxa in each network.

|  | **Treatment** | **ID** | **Phylum** | **Class** | **Order** | **Family** | **Genus** |
| --- | --- | --- | --- | --- | --- | --- | --- |
| **Module hub** | Endosphere (control) | ASV217 | Proteobacteria | Alphaproteobacteria | Sphingomonadales | Sphingomonadaceae | Sphingobium |
|  | Endosphere (SCMV) | ASV1121 | Bacteroidetes | Sphingobacteriia | Sphingobacteriales | Chitinophagaceae | Flavitalea |
| **Connector** | Rhizosphere (control) | ASV62 | Firmicutes | Clostridia | Clostridiales | Peptostreptococcaceae | Sporacetigenium |
|  | Rhizosphere (control) | ASV139 | Proteobacteria | Alphaproteobacteria | Rhizobiales | Bradyrhizobiaceae | Bradyrhizobium |
